# Effects of pharmacological modulation of cortical excitability on resting-state EEG PAC in humans

**DOI:** 10.1038/s41598-026-55712-5

**Published:** 2026-06-16

**Authors:** Alper Er, Paolo Belardinelli, Véronique Marchand-Pauvert, Ulf Ziemann, Guillaume Marrelec

**Affiliations:** 1 Sorbonne Université, CNRS, INSERM, Laboratoire d’Imagerie Biomédicale, LIB, 75006 Paris, France; 2 Università di Trento, Brain Plasticity Group - Center for Mind/Brain Science, CIMeC, 38123 Trento, Italy; 3 Hertie Institute for Clinical Brain Research, University of Tübingen, 72076 Tübingen, Germany; 4 Department of Neurology & Stroke, University of Tübingen, 72076 Tübingen, Germany

**Keywords:** E/I balance, Resting-state EEG, PAC, AMPA, NMDA, Neurology, Neuroscience

## Abstract

Electroencephalography (EEG) signal is driven by oscillations arising from the synchronized activity of cortical neuronal populations. Phase-amplitude coupling (PAC) refers to the interaction whereby the amplitude of higher-frequency oscillations is systematically modulated by the phase of slower rhythms. It has been suggested that the excitation/inhibition (E/I) balance, which is often disrupted in neuropsychiatric and neurodegenerative disorders, plays a critical role in shaping PAC, although direct evidence in humans remains limited. In this study, we investigated the effects of pharmacologically modulated E/I balance on PAC using resting-state EEG (rs-EEG) data. Specifically, we analyzed the impact of anti-glutamatergic drugs, dextromethorphan (NMDA receptor antagonist) and perampanel (AMPA receptor antagonist), as well as nimodipine (L-type voltage-gated calcium channel blocker) on PAC in a double blind, placebo-controlled experiment. Analysis revealed a significant decrease in PAC following administration of dextromethorphan and perampanel, while nimodipine and placebo showed no effects. By showing that PAC is sensitive to pharmacological modulation consistent with shifts in cortical E/I balance, this study confirms its physiological relevance and supports its use as a biomarker of excitability disturbances and cortical dysfunction across neurological and psychiatric disorders.

## Introduction

Electroencephalography (EEG) is a non-invasive technique that captures the brain oscillatory activity with high temporal resolution, enabling the characterization of neural dynamics across multiple frequency bands. Among the various notions derived from EEG signals, cross-frequency coupling (CFC) suggests that there might be an interplay between oscillatory activities at different frequencies. A specific form of CFC, known as phase-amplitude coupling (PAC), quantifies how the phase of a slower oscillation modulates the amplitude of a faster one, providing insight into the temporal coordination of neural activity across spatial scales^1^. Since neural oscillations reflect rhythmic fluctuations in excitability^2–4^, and communication between neuronal populations requires precise phase alignment across frequencies, PAC has been hypothesized to reflect the underlying balance between excitation and inhibition (E/I) in the cortex^5^.

Altered PAC has been increasingly reported across a range of neurological and psychiatric disorders characterized by disrupted E/I balance, although the underlying mechanisms of this disruption differ across conditions. In Parkinson’s disease, dopaminergic depletion alters basal ganglia–cortical circuit dynamics^6^. In epilepsy, E/I imbalance is typically driven by excessive excitation or impaired inhibition, often due to altered glutamatergic or GABAergic signaling, resulting in hypersynchronous neuronal activity^7,8^. In schizophrenia, converging evidence supports a glutamatergic hypothesis involving NMDA receptor hypofunction, particularly affecting parvalbumin-positive (PV+) interneurons, which disrupts inhibitory control and cortical synchrony^9,10^. In Alzheimer’s disease, synaptic dysfunction and amyloid-related alterations in inhibitory interneurons contribute to network hyperexcitability and impaired oscillatory coordination^11,12^. In ALS, cortical hyperexcitability has been associated with glutamatergic dysfunction, reduced inhibitory tone and altered noradrenergic neuromodulation^13,14^. An alteration of PAC has been reported across these various conditions, supporting the idea that PAC may represent a particularly sensitive marker of this shared network-level dysfunction, rather than reflecting disorder-specific mechanisms, and thus may serve as a candidate biomarker for neurophysiological alterations across neurological and psychiatric disorders.

Computational models demonstrate that one possible mechanism for PAC is the interplay between excitatory pyramidal neurons and distinct inhibitory interneuron classes^15–18^. Consistent with this mechanistic framework, in vivo and ex vivo experiments have shown that perturbations of inhibitory networks disrupt PAC: selective deletion of $\text {GABA}_{\textrm{A}}$ receptors from $\text {PV}^+$ interneurons markedly reduces theta–gamma coupling in mice^19^.

However, to date, the impact of E/I balance alterations on PAC in the human neocortex remains untested. Establishing this link is essential to validate PAC as a mechanistically grounded biomarker of cortical dynamics.

Pharmacological manipulation provides a robust experimental framework to probe E/I balance in a controlled manner. Among the key contributors to this balance is glutamatergic neurotransmission, which critically regulates neuronal excitability in the cerebral cortex^20^. Notably, disruptions in glutamatergic signaling are implicated in a range of neurological conditions, including schizophrenia^21^, epilepsy^22^, and ALS^23^.

In the present study, we investigated the relationship between PAC and E/I balance using a placebo-controlled, pharmacological EEG dataset involving the administration of anti-glutamatergic drugs perampanel and dextromethorphan, as well as a calcium channel blocker nimodipine. Perampanel^24^ is a selective, non-competitive antagonist of the *α*-amino-3-hydroxy-5-methyl-4-isoxazole propionic acid receptor (AMPAR), clinically used for the treatment of partial-onset and generalized tonic–clonic seizures. AMPARs are heteromeric ion channels typically composed of GluR1–4 subunits, with GluR2 being the most prevalent in the central nervous system. The presence of GluR2 renders the receptor channel impermeable to calcium, whereas GluR2-lacking receptors are calcium-permeable and predominantly expressed in interneurons and selected cortical neurons. Perampanel inhibits all AMPAR subunits with similar potency, thereby attenuating fast excitatory neurotransmission mediated by sodium influx through GluR2-containing AMPARs, which are characterized by rapid and temporally precise kinetics. Dextromethorphan^25^ by contrast, acts as a non-competitive antagonist of the N-methyl-D-aspartate receptor (NMDAR), which is highly permeable to calcium and plays a pivotal role in synaptic plasticity, particularly in the induction of long-term potentiation (LTP). NMDAR activation requires both glutamate binding and co-activation by glycine. In addition to its NMDA-blocking action, dextromethorphan exhibits broader pharmacological activity, including agonist effects at Sigma-1 receptors, competitive inhibition of serotonin reuptake, and weaker interactions with noradrenaline transporters. Finally, nimodipine^26^, is a dihydropyridine-class antagonist of L-type voltage-gated calcium channels (L-VGCCs), primarily used in the treatment of cerebral vasospasm following subarachnoid hemorrhage. These channels mediate calcium influx into presynaptic neurons and, together with NMDARs, contribute critically to activity-dependent glutamate release. By blocking L-VGCCs, nimodipine effectively diminishes calcium-dependent neurotransmitter release and modulates synaptic excitability. Extending previous work that examined the effects of these drugs on transcranial magnetic stimulation-EEG (TMS-EEG) responses^27^, the present study focuses on resting-state EEG (rs-EEG) to assess PAC modulation between post- and pre-drug administration. Based on the known pharmacological profiles of these compounds, we hypothesized that AMPAR and NMDAR antagonism (perampanel and dextromethorphan) would modulate PAC by shifting cortical E/I balance toward reduced excitation, whereas nimodipine, primarily affecting calcium-dependent neurotransmitter release rather than postsynaptic receptor activation, would produce minimal or no systematic PAC changes. In contrast, the placebo condition was not expected to induce any measurable modulation of PAC.

## Material and methods

**Fig. 1:**
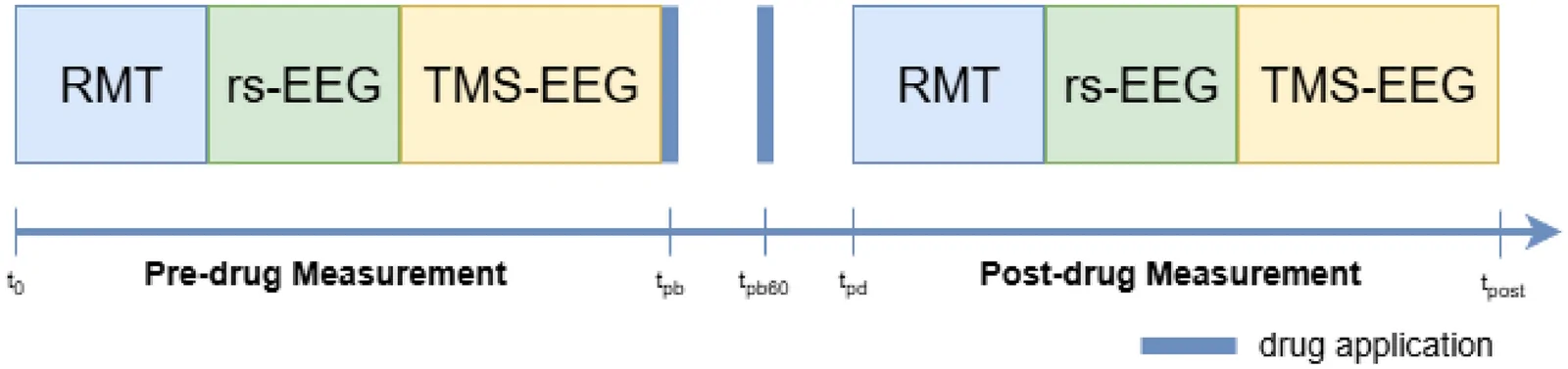
Experimental procedure. Time markers: t_0_ *=* pre-measurement, t_pb_ *=* first time-marker for drug application, t_pb60_ *=* 60 min after t_pb_ for second drug application, t_pd_ *=* 120 min post first drug intake, t_post_ *=* time-marker after post-drug measurement.

The data used in the present work are part of an experiment that was partly used in a previous publication^27^. While the previous publication exclusively focused on the TMS-EEG part of the experiment, we here analyzed PAC modulation in the rs-EEG (see Fig. 1 below).

### Participants

Eighteen healthy male volunteers (mean age: 26.0 years, range: 24–36 years) participated in our study after providing written informed consent. Inclusion criteria were right-handedness with a laterality score *>75%* according to the Edinburgh handedness inventory^28^ and male gender to avoid an impact of the menstrual cycle on cortical excitability^29^. Exclusion criteria were past or present somatic, neurological or psychiatric disorders, need for continuous medication, use of recreational or illicit drugs (including nicotine and alcohol), presence of any contraindication to TMS^30^ and MRI, or to the study medication. Study included blood pressure screening to exclude patients with hypotension (*<90/60* mmHg according to the US National Heart, Lung, and Blood Institute)^31^. Furthermore, subjects with resting motor threshold (RMT) *>60%* were excluded to minimize EEG artefacts (see below). Participants were advised not to conduct vehicles for seven days after each session due to the half-time of the study medications.

All experimental procedures were carried out in accordance with relevant guidelines and regulations and with the latest revision of the Declaration of Helsinki. The study was approved by the ethics committee of the medical Faculty of Eberhard-Karls-University Tübingen (Project No. 526/2014BO1). Written informed consent was obtained from all participants prior to participation.

### Experimental design

A pseudorandomized, double-blinded, placebo-controlled crossover pharmaco–TMS–EEG study was conducted in healthy human subjects with the following medications: perampanel, dextromethorphan, nimodipine and placebo. The study consisted of four sessions for each condition, with an interval of at least two weeks between each session to avoid carry-over effects of study medications. The approval of the study and study medication, including dosage and use, was made by the ethics committee of the medical Faculty of Eberhard-Karls-University Tübingen (Project No. 526/2014BO1).

### Experimental procedure

Each experimental session consisted of two phases: a pre-drug and a post-drug measurement. The timeline of a typical session is illustrated in Fig. 1. Each phase included three blocks: (1) identification of the motor hotspot, the scalp position that elicited the largest and most consistent response in the target muscle, and assessment of RMT, (2) recording of rs-EEG, and (3) TMS–EEG signal acquisition.

Drug administration occurred between the pre- and post-drug phases, with two time points labeled t_pb_ and t_pd60_. Based on prior pharmacokinetic studies (Table 1), post-drug measurements were scheduled to coincide with the expected peak plasma concentration (T_max_) of each compound. Specifically, a 1-hour delay was used for perampanel (based on unpublished data from Eisai Co. Ltd.), 2 hours for dextromethorphan^32^, and 1 hour for nimodipine (Nimotop® product information).

EEG recordings were performed using a 62-channel cap with c-ring electrodes arranged according to the international 10–20 system (BrainCap-Fast’n Easy; Brain Products), connected to a TMS-compatible EEG amplifier (BrainAmp DC; Brain Products). Electrode impedance was maintained below 5 k*Ω* throughout the session by preparing the scalp with an abrasive gel (Nuprep®Skin Prep Gel, Weaver and Company, USA) and applying conductive cream (Hellige Conductive Cream, GE Healthcare, USA).

To evaluate drug effects on spontaneous brain activity, three minutes of rs-EEG was recorded with eyes open both before and after drug administration. During these recordings, participants sat comfortably and were instructed to fixate on a spot marked on the wall in front of them.

The present study is restricted to rs-EEG analysis, but a detailed description of the TMS–EEG procedures can be found in a previous publication^27^.

**Table 1:** Pharmacokinetics and drug administration. Dosages, administration times, and expected onset times for each drug and placebo are given. To ensure double-blinding, placebo capsules were administered at t_pb_ due to the reported onset time of dextromethorphan; at t_pb60_, perampanel and nimodipine tablets were administered. (*) A dose of 12 mg perampanel was initially administered to the first three participants. However, one of them experienced adverse effects (dizziness, nausea, and ataxia), prompting a reduction of the dose to 6 mg for the remaining 13 participants.

Drug and Brand	Dosage	$T_{\max }$ [h]	Application at t_pb_	Application at t_pb60_
**Perampanel** (Fycompa®, Eisai Co Ltd)	12 mg or 6 mg (*)	0.5–2.5	Placebo	**Perampanel**
**Dextromethorphan** (Hustenstiller-ratiopharm®)	120 mg	1–2	**Dextromethorphan**	Placebo
**Nimodipine** (Nimodipin-HEXAL®)	30 mg	0.6–1.6	Placebo	**Nimodipine**
**Placebo** tablets (P-Tabletten Lichtenstein, 7, 8, 10 mm, Winthrop)	—	—	Placebo	Placebo

### EEG processing

#### Pre-processing

Data were preprocessed using customized Matlab scripts (Matlab R2016a, The Mathworks) and the open source toolbox Fieldtrip^33^. To investigate the effect of the drugs on oscillatory activity, resting-state data were segmented in epochs of 2 s, filtered with a 1–80 Hz bandpass filter (zero-phase Butterworth, 3rd order) and a 49–51 Hz notch filter (zero-phase Butterworth, 3rd order) and downsampled to 1000 Hz. Epochs containing major artifacts and noisy EEG channels were removed upon visual inspection (percentage of removed epochs for all pre/post-drug conditions: *14.6 ± 8.9*%, number of removed channels: *5± 2*; means ± 1 s.d.). ICA components representing biological (eye blinks and movements, persistent muscle activity) or electrical artifacts were removed on the basis of their topography, single-trial time-course and power spectrum (components removed: 18±4). Then, discarded channels were spline-interpolated using the signal of the neighboring channels^34^ and data were re-referenced to the average reference signal. Based on data quality, the final number of subjects included in the analysis was as follows: 16 for perampanel, 15 for dextromethorphan, 14 for nimodipine, and 16 for the placebo condition. To ensure consistency across participants and conditions, we standardized the number of epochs used in the analysis. Specifically, we retained the first 35 good epochs per recording, corresponding to the minimum number of usable epochs available across all subjects (in either the pre- or post-intervention phase).

#### Spectral power analysis

We performed spectral power analysis across the four canonical frequency bands: theta (4–8 Hz), alpha (8–12 Hz), beta (13–30 Hz), and gamma (30–60 Hz). Power spectral density (PSD) was computed on each epoch using a multitaper method^35^ over the 2–70 Hz frequency range and averaged across epochs to obtain a stable spectral estimate per channel. To decompose each power spectrum into its aperiodic and periodic components, we applied the FOOOF algorithm^36^ with the knee aperiodic mode, fitted over the 2–70 Hz range using the following parameters: peak width limits of 1–8 Hz, a maximum of 6 peaks, and a minimum peak height of 0.1. Model fit was monitored using the $R^2$ between each observed spectrum and its estimated counterpart. Only spectra with a $R^2$ larger than 0.8 were used for the subsequent spectral power analysis, while others were discarded. The three aperiodic parameters yielded by the model fit, offset, exponent, and knee, were extracted and carried forward for statistical analysis. For spectra with a good fit, the modeled aperiodic component was then subtracted from the power spectrum to yield an aperiodic-corrected residual spectrum. For each channel, the aperiodic-corrected power was integrated across frequency bins within each predefined band using the trapezoidal method, yielding a measure of total periodic power above the aperiodic baseline.

#### PAC analysis

We carried out PAC analysis using a custom Python script based on the Pactools library^37^. The analysis was performed for each EEG channel, with PAC computed separately for each epoch and then averaged across all epochs to obtain a stable estimate.

PAC was calculated between the phase of the theta band (4–8 Hz) and the amplitude of the gamma band (30–60 Hz). Both frequency ranges were discretized into 30 values, yielding a *30 × 30* frequency pair grid. Signals were band-pass filtered around each frequency value using a 2 Hz bandwidth for the low-frequency range and a bandwidth equal to twice the maximum low-frequency value (i.e., *2 × 8 = 16* Hz) for the high-frequency range^38,39^. The Hilbert transform was applied to extract the instantaneous phase of the low-frequency components and the amplitude envelope of the high-frequency components. PAC strength was then quantified using the Modulation Index (MI) method^40^, the most widely accepted robust measure of PAC^41^. This procedure produced a *30 × 30* matrix, also called comodulogram, representing the coupling between each low-frequency phase and high-frequency amplitude pair.

Each comodulogram was then averaged across both its low- and high-frequency ranges to yield a single PAC value per epoch. Epoch-specific values were then averaged across all epochs per subject and projected onto the EEG scalp layout to produce topographical PAC maps. These spatial representations were used in the statistical comparisons between conditions.

### Statistics

Statistical analyses were performed in Python using functions from the MNE^42^ and SciPy^43^ libraries. A significance threshold of *p < 0.05* was applied for all statistical tests.

To evaluate spatial changes in aperiodic parameters, spectral power, and PAC, both for verifying pre-drug comparability across conditions and for assessing pre- versus post-drug changes, we employed a non-parametric cluster-based permutation approach as described by Oostenveld et al.^44^. For the pre-drug comparison across conditions, the Friedman test was used as the cluster-forming statistic, which is the non-parametric equivalent of a one-way repeated measures ANOVA, and only subjects with valid data across all conditions were included to ensure a consistent sample. For the pre- versus post-drug comparison, given the within-subject paired design, we used the paired Wilcoxon signed-rank test as the cluster-forming statistic instead of the more commonly used paired t-test, following recommendations of Candia et al.^45^. Unlike the paired *t*-test, which yields a *t* statistic that is symmetrically distributed around zero under the null hypothesis, such that larger positive or negative values indicate stronger deviations from the null hypothesis, the Wilcoxon signed-rank test produces a *W* statistic such that smaller *W* values reflect stronger deviations from the null hypothesis. This inversion complicates the direct comparison of test statistics across clusters and their visualization in topographic maps. To make the interpretation consistent with the conventional cluster-permutation framework (where higher values indicate stronger effects), we first transformed the raw *W* values into *z*-scores to account for distributional differences and scale them to a common metric. We then flipped the sign of the standardized values, so that larger *z*-scores now correspond to stronger effects, analogous to the behavior of the *t* statistic. This approach allowed us to identify electrode clusters exhibiting consistent condition-related changes, while controlling for multiple comparisons through a non-parametric framework. For a significant cluster identified in the topographical analysis, we extracted the mean PAC value across all electrodes within the cluster for each subject for the further post-hoc analysis with the paired Wilcoxon signed-rank test and also for the boxplot representation.

## Results

### Spectral power analysis

**Fig. 2:**
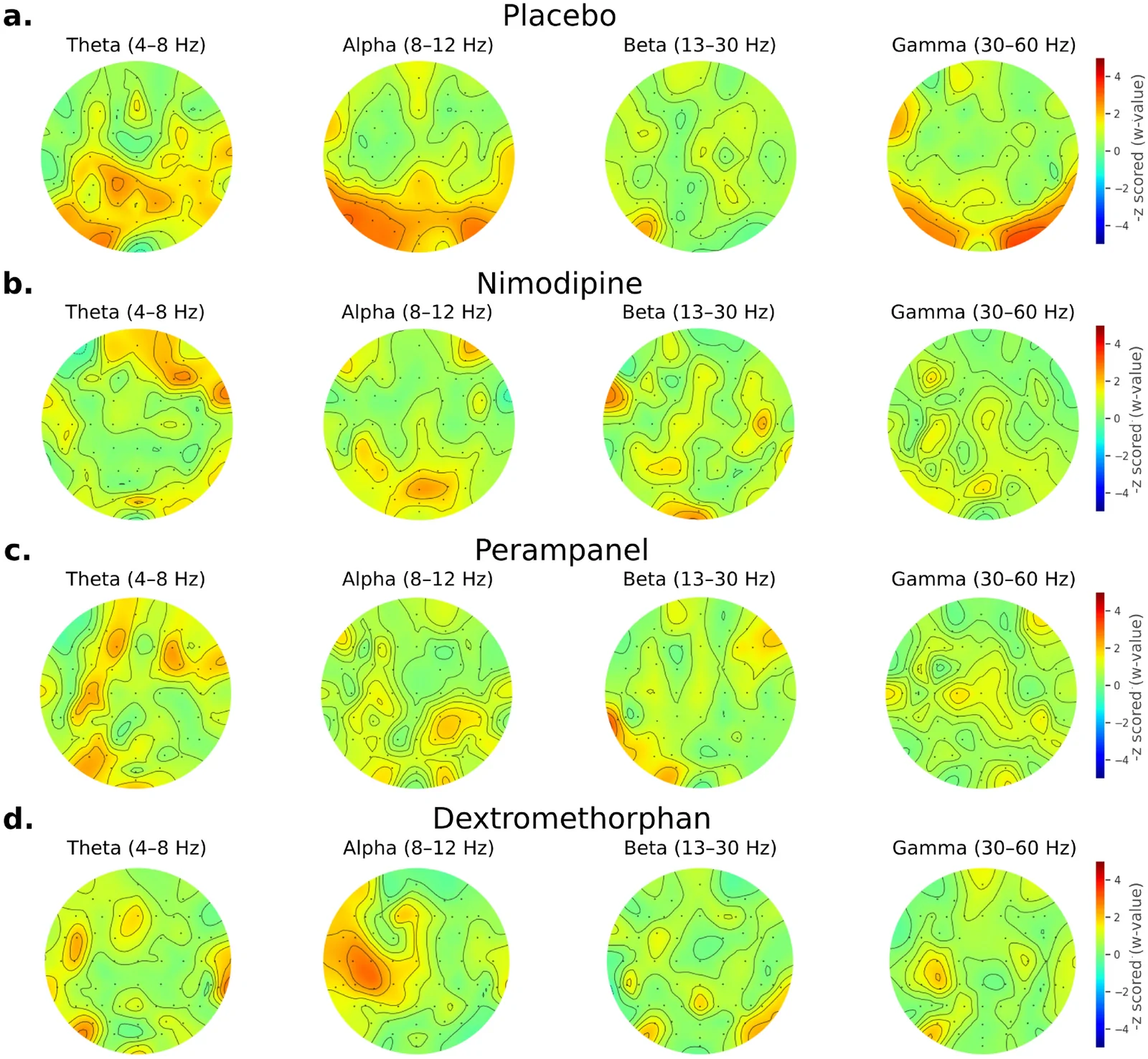
Results from spectral power analysis. Post minus pre comparisons using the paired Wilcoxon signed-rank test combined with cluster-based permutation analysis across the canonical frequency bands: theta, alpha, beta, and gamma. Results are shown for each condition: placebo (**a**), nimodipine (**b**), perampanel (**c**), and dextromethorphan (**d**). Figures were generated using Python 3.10 (https://www.python.org/) with custom functions based on the MNE toolbox (https://mne.tools), and visualized using the Matplotlib (https://matplotlib.org/) and Seaborn (https://seaborn.pydata.org/) libraries.

Following analysis with FOOOF, two subjects of the dextromethorphan condition were excluded due to poor model fit ($R^2 =$ 0.49 and 0.56, respectively); these subjects were retained in all other conditions (placebo, nimodipine, and perampanel). Pre-drug recordings revealed no significant differences in aperiodic parameters (offset, exponent, and knee) or spectral power across conditions, confirming that the groups were comparable prior to drug administration. Cluster-based permutation tests on pre- versus post-drug recordings revealed no significant differences in aperiodic parameters across any condition. Similarly, no significant changes in spectral power were observed across any condition, including placebo, nimodipine, perampanel, and dextromethorphan (Fig. 2a–d).

### PAC analysis

Based on topographical cluster-based permutation testing, pre-drug recordings showed no significant differences in PAC between conditions, confirming comparability prior to drug administration.

Figure 3 presents the results of the PAC analysis for each condition: placebo (Fig. 3a), nimodipine (Fig. 3b), perampanel (Fig. 3c) and dextromethorphan (Fig. 3d). No significant PAC differences were observed following placebo or nimodipine administration. Statistically significant clusters were identified for both the perampanel and dextromethorphan conditions, localized over a left fronto-central topography and a right centro-parietal topography, respectively. For the dextromethorphan condition, the analysis was performed on *N = 13* subjects following the exclusion of two subjects due to poor FOOOF model fit. Notably, when retaining all *N = 15* subjects, the same right centro-parietal cluster was recovered with a loss of a single electrode (Cb6), indicating that the exclusion had minimal impact on the topographical results. The corresponding boxplots below panel (c) and panel (d) illustrate the mean PAC values within the clusters before and after drug administration, demonstrating a significant post-drug decrease for both drugs (paired Wilcoxon signed-rank test).

**d 3:**
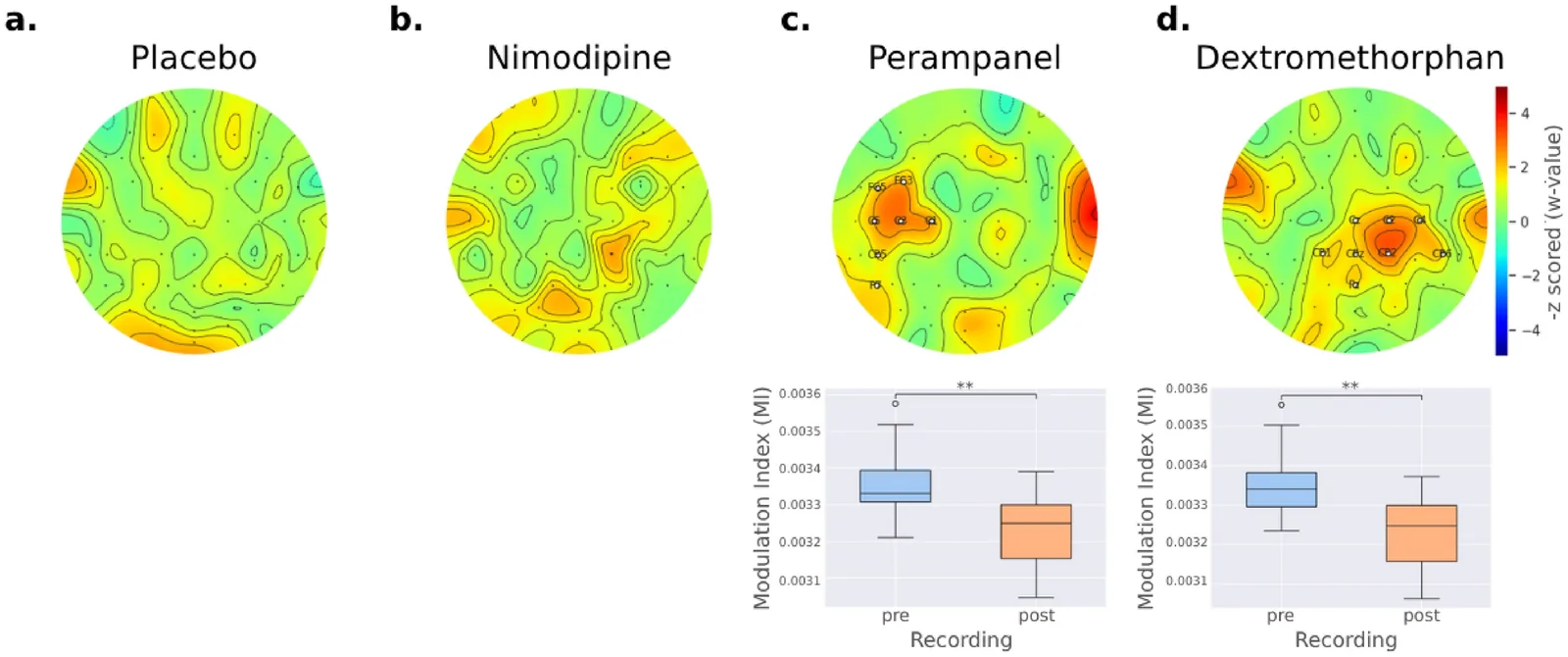
PAC results for all conditions. Post minus pre comparisons using Wilcoxon signed-rank test combined with cluster-based permutation analysis are shown for each condition: placebo (**a**), nimodipine (**b**), perampanel (**c**), and dextromethorphan (**d**). Below panels (**c**) and (**d**), boxplots display the mean PAC values within the significant clusters before and after drug administration. Figures were generated using Python 3.10 (https://www.python.org/) with custom functions based on the MNE toolbox (https://mne.tools), and visualized using the Matplotlib (https://matplotlib.org/) and Seaborn (https://seaborn.pydata.org/) libraries. ** : *p < 0.01*.

## Discussion

In this study, we investigated whether pharmacological modulation of glutamatergic neurotransmission alters PAC in rs-EEG recordings of the human cortex. Specifically, we examined the effects of three compounds, perampanel (an AMPA receptor antagonist), dextromethorphan (an NMDA receptor antagonist), and nimodipine (a calcium channel blocker), in a placebo-controlled crossover design. Here, we did not find any significant power changes in 1/f-corrected spectra across any of the drug conditions. In parallel, theta–gamma PAC was significantly reduced following the administration of both drugs, with distinct topographical patterns: a left fronto-central cluster for perampanel and a right centro-parietal cluster for dextromethorphan. By contrast, neither nimodipine nor placebo produced significant changes in PAC.

The pharmacological agents used in this study were chosen for their distinct effects on cortical E/I balance, primarily through modulation of glutamatergic signaling and neuronal excitability. Perampanel, a selective non-competitive AMPA receptor antagonist, reduces fast excitatory synaptic transmission by blocking AMPA-mediated currents, thereby shifting the E/I balance toward inhibition^24^. Dextromethorphan, an NMDA receptor antagonist, suppresses slower glutamatergic transmission by inhibiting NMDA-dependent depolarization^25^. Nimodipine, a blocker of L-type voltage-gated calcium channels, acts more indirectly by reducing calcium influx, which can lower both presynaptic glutamate release and postsynaptic excitability^46^. Together, these compounds offer complementary approaches to experimentally alter the E/I balance in the human cortex.

**Baseline (pre-drug) recordings across all conditions showed no significant differences** in spectral power or PAC, indicating comparable pre-intervention neural activity and supporting the specificity of the observed drug-induced effects.

**Placebo administration did not produce any significant changes** in either spectral power or PAC, supporting that the observed effects were specific to active pharmacological modulation rather than non-specific experimental or temporal factors.

The absence of significant spectral power changes in our data contrasts with several previous reports. For perampanel, MEG studies have reported elevated low-frequency power following AMPA receptor blockade^47^, and an EEG study in epileptic patients found increased theta but decreased alpha power after six months of treatment^48^. Increases in theta power have also been linked to perampanel-induced sleepiness^49^, a side effect not reported by any participant in our study. For dextromethorphan, preclinical work has shown that ketamine enhances gamma oscillations, and increases in alpha power alongside reductions in theta activity have been frequently reported ^50–52^. It is worth noting that a significant cluster of increased alpha power would be observed in case of dextromethorphan when keeping the two subjects excluded for poor fit. More broadly, discrepancies between our findings and the literature may also reflect methodological differences across studies, including recording modality, patient population, treatment duration, and the resting-state nature of our paradigm. Additionally, none of these studies explicitly report whether aperiodic correction was applied prior to spectral analysis. Given that broadband power changes can largely reflect shifts in the aperiodic component, this methodological difference may partly account for the discrepancies observed between our findings and the literature.

**Reduced theta–gamma PAC following perampanel administration over fronto-central regions** provides novel evidence that PAC is sensitive to pharmacological modulation of excitatory transmission in the human brain. AMPA receptor antagonists such as perampanel are well known to reduce fast glutamatergic signaling and suppress cortical excitability, yet their impact on large-scale oscillatory coordination has remained largely uncharacterized. Our results suggest that theta–gamma PAC may serve as a sensitive index of such modulation, likely reflecting disrupted coupling between slower integrative theta rhythms and faster local gamma activity. To our knowledge, no previous study in either human or preclinical models has directly linked AMPAR blockade to selective PAC suppression, positioning our findings as the first to demonstrate that reduced excitatory drive via perampanel alters cross-frequency coupling dynamics at rest.

**Dextromethorphan-induced PAC reduction over centro-parietal areas** may reflect a complex interaction between NMDA receptor hypofunction and cortical E/I balance. This finding is particularly relevant for disorders such as schizophrenia, where NMDA receptor hypofunction and E/I imbalance are central to pathophysiology^9,10^. In this context, the reduction in PAC observed following NMDA receptor antagonism in our study could provide a mechanistic parallel supporting the use of PAC as a translational biomarker of cortical dysfunction. Preclinical studies offer partial parallels: for instance, the NMDA receptor antagonist MK-801 has been shown to impair theta–gamma coupling, suggesting that shifts in E/I balance can selectively disrupt temporal coordination^53^. Conversely, ketamine has demonstrated dose-dependent effects, enhancing theta–gamma PAC at low doses but disrupting it at higher doses, likely due to network-level desynchronization and hyperexcitability^54^. These findings suggest a non-linear relationship between NMDA receptor function and PAC, where cross-frequency coupling is maintained only within a narrow window of balanced excitation and inhibition. At the cellular level, this vulnerability may arise from the role of (PV+) interneurons, which depend on NMDA receptor activation to provide fast, precisely timed inhibitory control. Selective NMDA receptor ablation in PV+ interneurons leads to increased gamma power but reduced theta–gamma modulation and impaired synchrony^50^, highlighting the importance of intact inhibitory timing for PAC generation. Within this framework, dextromethorphan may alter E/I balance by pushing the system beyond a threshold of stable inhibition, leading to disrupted phase-amplitude coordination. This hypothesis is also in line with the findings in ALS where the decrease in theta–gamma PAC was associated to the hyperexcitability in a translational study connecting pre-clinical LFP recordings and clinical EEG recordings ^13^.

Previous results demonstrated that dextromethorphan increases the N45 component of TMS-evoked potentials (TEPs)^27^, which is similarly enhanced by GABAergic agonists like benzodiazepines^55^, suggesting that NMDA receptor antagonism shift cortical excitability towards inhibition. This challenges a purely disinhibitory interpretation and instead points toward a broader disruption in E/I coordination. Supporting this interpretation, theta–gamma PAC was also reduced following administration of perampanel, an AMPA receptor antagonist that decreases excitatory drive.

This discrepancy between the preclinical interpretation of NMDA blockade as inducing hyperexcitability and our observation of reduced PAC may be explained by differences in recording modalities and spectral definitions. While preclinical studies typically use high-SNR intracranial LFP recordings and broader gamma or high frequency oscillation (HFO) ranges (e.g., theta: 4–12 Hz; gamma: 30–80 Hz; HFOs: 120–160 Hz), our scalp EEG analysis was restricted to narrower bands (theta: 4–8 Hz; gamma: 30–60 Hz), possibly making our PAC estimates more sensitive to desynchronization and less reflective of localized hyperexcitability. From another perspective, methodological differences in signal processing and PAC estimation may represent a major source of variability across studies^39^.

**Both perampanel and dextromethorphan reduced PAC**, suggesting that blocking glutamatergic transmission, whether through AMPA or NMDA receptors, converges on a common disruption of cross-frequency coordination. These effects emerged over distinct topographic clusters: a centro-frontal cluster for perampanel and a centro-parietal cluster for dextromethorphan. The main presence of PAC modulation focalisation around central electrodes can be explained by sensorimotor activation in rs-EEG^56^. It should be noted that scalp EEG topographies provide only indirect spatial information, and the limited spatial resolution of surface recordings does not permit precise cortical localization or strong region-specific functional conclusions. With this caveat in mind, the topographic dissociation between the two drugs remains an interesting observation that may hint at their distinct functional mechanisms. Tentatively, the fronto-central distribution observed with perampanel might suggest a greater susceptibility of frontal networks to AMPA blockade, possibly reflecting disruption of rapid, transient signaling involved in executive and dynamic routing processes. Similarly, the centro-parietal distribution observed with dextromethorphan might point toward an impairment of slower integrative processes associated with sustained sensory representation, characteristic of parietal computation. However, these regional observations should be considered as hypothesis-generating rather than conclusive, and would require source-level analyses or intracranial recordings to be properly evaluated. Differential effects were also reflected on TEPs: perampanel selectively modulates the P60 component, while dextromethorphan alters the N45 in different clusters^27^. This is consistent with prior evidence that AMPA and NMDA receptor antagonists exert differentiated effects on synaptic and circuit-level activity, as shown in both neurophysiological and neuroimaging studies^57^. Taken together, these findings suggest that AMPA and NMDA receptor antagonists target complementary aspects of glutamatergic transmission. Nonetheless, the convergence in PAC suppression suggests that both receptor systems are critically involved in the temporal integration of oscillatory activity, reinforcing the idea that glutamatergic transmission plays a fundamental role in maintaining the coordination of neural dynamics in the resting brain.

**Dissociation between unchanged spectral power and decreased PAC** observed for both perampanel and dextromethorphan highlights the distinction between oscillatory amplitude and cross-frequency coordination. While spectral power reflects the strength of local oscillatory activity, PAC captures the dynamic coupling between slower and faster rhythms, an index of hierarchical organization and temporal precision. Notably, neither perampanel nor dextromethorphan produced significant changes in gamma band power, yet both drugs led to a significant reduction in theta–gamma PAC. While one might expect a direct interaction between gamma band power and theta–gamma PAC given that gamma amplitude is a core component of PAC estimation, the absence of significant gamma power changes alongside a consistent PAC decrease indicates that the observed PAC reduction cannot be attributed to changes in gamma amplitude. This dissociation suggests that PAC captures aspects of neural organization that are not readily visible to spectral power analysis alone, and that the PAC reduction should reflect a genuine disruption of cross-frequency coordination itself, independent of power-related confounds. This interpretation is consistent with prior work showing that theta–gamma PAC can decrease independently of gamma power^50,54,58^. Together, these results provide a proof of concept that PAC may constitutes a complementary metric to spectral power, capable of capturing drug-induced alterations in cortical dynamics that are not obvious from power measures alone.

**Absence of PAC change following nimodipine administration**, an L-type voltage-gated calcium channel (L-VGCC) blocker, was in line with previous results sowing no effect on TEPs and TMS-induced oscillations (TIOs)^27^. A dose-related explanation is unlikely, as the same 30 mg oral dose has previously suppressed LTP- and LTD-like plasticity in human motor cortex^59^. Moreover, nimodipine has also shown minimal effects on the MEP recruitment curve^60^. Taken together, these findings indicate that the absence of PAC modulation under nimodipine likely reflects the fact that L-VGCCs contribute only indirectly to cortical excitability. L-type channels are predominantly located on dendrites and require strong postsynaptic depolarization to open, thereby playing a limited role in fast glutamatergic transmission and synaptic integration at rest. This pharmacological profile is consistent with the lack of effects observed here on resting-state PAC, in contrast to the direct modulation of glutamatergic receptors induced by dextromethorphan and perampanel.

Differently from our previous work which detected no effect of these drugs on TMS-induced oscillations with a standard approach (Belardinelli et al.^27^, Fig. 5) and only some mild effects of perampanel with PARAFAC (Belardinelli et al.^27^, Fig. 7), PAC appears a more sensitive metric to reveal oscillations-related changes due to glutamatergic drug assumption.

In this study, the comodulogram, which represents a two-dimensional frequency-frequency map of coupling strength, was summarized into a single scalar value per epoch by averaging across all theta–gamma frequency pairs. This dimensionality reduction offers several advantages: it limits the number of statistical comparisons, mitigates noise-related variability, and results in more stable and reliable PAC estimates given the limited number of epochs available per recording. It should nonetheless be acknowledged that reducing the comodulogram to a single scalar does not preserve the frequency-specific coupling structure of the two-dimensional representation, and may therefore obscure fine-grained differences in coupling patterns within the theta–gamma range. Among the possible summary statistics, averaging was preferred over taking the maximum value across frequency pairs, as the maximum is more susceptible to spurious isolated peaks and epoch-level variability in short recordings, and risks reflecting local noise rather than a consistent and stable coupling pattern across the full theta–gamma range.

## Conclusion

Pharmacological modulation of glutamatergic transmission with both AMPA and NMDA receptor antagonists leads to a reduction in theta–gamma PAC without affecting spectral power. This dissociation underscores the complementary value of PAC as a sensitive marker of cortical network dynamics that captures alterations in temporal coordination not apparent from power measures alone. The convergent suppression of PAC by perampanel and dextromethorphan indicates that intact glutamatergic signaling through both receptor systems is essential for sustaining oscillatory interactions, highlighting PAC as a promising non-invasive biomarker sensitive to pharmacological perturbations of cortical excitability that are consistent with E/I balance shifts, in human rs-EEG recordings.

## Data Availability

The data supporting the findings of this study are not publicly available due to ethical and data protection considerations related to the experimental protocol, but may be made available for scientific purposes upon reasonable request and subject to approval by Prof. Ulf Ziemann.
